# Modelling the cost differential between healthy and current diets: the New Zealand case study

**DOI:** 10.1186/s12966-018-0648-6

**Published:** 2018-02-09

**Authors:** Stefanie Vandevijvere, Nick Young, Sally Mackay, Boyd Swinburn, Mark Gahegan

**Affiliations:** 1 School of Population Health, Department of Epidemiology and Biostatistics, The University of Auckland, Private Bag 92019, Auckland, 1142 New Zealand; 2 Centre for e-Research, Faculty of Science, The University of Auckland, Auckland, New Zealand

**Keywords:** Cost, Healthy diets, Food prices, Modelling, INFORMAS, Ethnic diets

## Abstract

**Background:**

Evidence on whether healthy diets are more expensive than current diets is mixed due to lack of robust methodology. The aim of this study was to develop a novel methodology to model the cost differential between healthy and current diets and apply it in New Zealand.

**Methods:**

Prices of common foods were collected from 15 supermarkets, 15 fruit/vegetable stores and from the Food Price Index. The distribution of the cost of two-weekly healthy and current household diets was modelled using a list of commonly consumed foods, a set of min and max quantity/serves constraints for each, and food group and nutrient intakes based on dietary guidelines (healthy diets) or nutrition survey data (current diets). The cost differential between healthy and current diets was modelled for several diet, prices and policy scenarios. Acceptability of resulting meal plans was validated.

**Results:**

The average cost of healthy household diets was $27 more expensive than the average cost of current diets, but 25.8% of healthy diets were cheaper than the average cost of current diets. This cost differential could be reduced if fruits and vegetables became exempt from Goods and Services Tax. Healthy diets were cheaper with an allowance for discretionary foods and more expensive when including takeaway meals. For Māori and Pacific households, healthy diets were on average $40 and $60 cheaper than current diets due to large energy intakes. Discretionary foods and takeaway meals contributed 30-40% to the average cost of current diets.

**Conclusion:**

Healthy New Zealand diets were on average more expensive than current diets, but one-quarter of healthy diets were cheaper than the average cost of current diets. The impact of diet composition, types of prices and policies on the cost differential was substantial. The methodology can be used in other countries to monitor the cost differential between healthy and current household diets.

**Electronic supplementary material:**

The online version of this article (10.1186/s12966-018-0648-6) contains supplementary material, which is available to authorized users.

## Background

Unhealthy diets contribute to increasing obesity and diet-related non-communicable diseases (NCDs) [1]. The cost of food is a major determinant of food choices [2, 3]. Some countries have implemented health-related taxes or subsidies in an effort to improve population diets [4]. Taxes on sugar-sweetened beverages (SSB) are increasingly common internationally [5], and have shown to significantly reduce SSB purchases, especially in lower socio-economic population groups [6, 7]. In addition, some countries do not tax healthy foods, for example, in Australia, there is no Goods and Services Tax (GST) on basic healthy foods [8].

Evidence on whether or not healthier diets or dietary patterns are more expensive than less healthy diets is mixed [9]. Currently there is no robust methodology available to adequately answer that question. Most previous studies did not sufficiently take into account the variation in possible diets or food prices, or measured only the cost of the healthy diet [9]. Healthy diets are usually based on food-based dietary guidelines, and are developed by substituting items in a typical diet with healthier items, by developing a diet to meet food-based dietary guidelines or based on a typical diet of those who meet dietary guidelines. Some studies used linear programming to develop healthy diets that meet nutrition recommendations for a minimum cost [10]. There are a few previous studies that did measure the cost differential between healthy and current diets, but they compared the cost of only one healthy diet with one current, less healthy population diet [11–15]. The variation of the cost of diets is important when considering the relative cost differential between healthy and current diets, but is currently unknown. Many diet scenarios can be constructed using a list of commonly consumed foods to meet nutrient and food-based dietary guidelines (for ‘healthy’ diets) or specified population nutrient and food intakes (for ‘current’ diets). The International Network for Food and Obesity/NCDs Research, Monitoring and Action Support (INFORMAS) [16] developed a useful framework to monitor the cost differential between healthy and current population diets globally [17]. Such monitoring aims to provide robust data and benchmarks to inform economic and fiscal policy responses.

Within the INFORMAS framework for monitoring the cost of population diets, this study developed a new tool and methodology, DIETCOST, and modelled, for the first time, the cost differential between current household diets and healthy household diets in New Zealand, taking into account the variations in two-weekly household meal plans and food prices. The cost differential between healthy and current household diets was compared between different ethnic population groups and for a series of diet and prices scenarios. In addition, the potential impact of two policy scenarios (fresh and frozen vegetables exempt from GST and a tax on SSBs) on the cost differential was modelled, and the Food Price Index (FPI) data was used to model the cost of current New Zealand household diets over a ten year period.

## Methods

The study was approved by the Human Participants Ethics Committee of the University of Auckland (ref 12330). A novel DIETCOST programme [18] was developed for researchers, using Python, to model the cost of healthy and current household diets using a list of commonly consumed foods, a set of min and max quantity/serves constraints for each, and specified food group and nutrient intakes based on dietary guidelines (healthy diets) and nutrition survey data (current diets for different population groups).

The programme was applied in New Zealand as a case study for the total population and specific ethnic population groups.

The reference household comprised a 45-year old man, a 45-year old woman, a 14-year old boy and a 7-year old girl.

### Inputs

The following inputs were prepared as Excel files:

- List of commonly consumed foods

The lists of common foods for the total population and the different ethnic population groups (Māori and Pacific populations) were derived from the New Zealand adult nutrition survey and the New Zealand children’s food and drink survey [19–22]. Foods within the different groups were included if consumed by more than 5% of the population. The resulting common foods list for Māori and Pacific populations was additionally checked by experts from Toi Tangata (for Māori) and Pacific Heartbeat (for Pacific) and a few items were deleted or added. Some foods were only included for children or only for adults dependent on consumption frequencies from surveys and advice from the expert panels. The final common foods list contained about 120-133 food and takeaway items dependent on the population group.

- Nutrition targets and constraints

The energy requirement for healthy adult diets was calculated using the Body Weight Calculator [23] based on a weight derived from a Body Mass Index (BMI) of 23 kg/m^2^, a mean population height [24], and moderate physical activity. The energy requirement for healthy children’s diets was based on the recommended energy requirements per KJ/kg per day from FAO/WHO/UNU [25] for moderate physical activity. The target weight was calculated using the 50th percentile BMI from the CDC growth charts [26] using mean height [24]. The energy requirement for current adult diets was based on the current BMI and moderate physical activity as over half of New Zealand adults meet the physical activity guidelines [24]. The energy requirement for current children’s diets was based on actual weight [24] and moderate physical activity as most children meet the New Zealand physical activity guidelines [27]. The additional energy required for the actual weight was calculated using a validated equation [28] for the excess energy intake per unit excess weight in childhood.

The daily food group and nutrient targets included serves of fruit, starchy and non-starchy vegetables, dairy, protein sources and grains, percentage of energy from fats, saturated fats, carbohydrates, protein, and total sugars, amount (g/mg) of fibre, red meat and sodium, and for certain scenarios the percentage of energy from alcohol and/or discretionary foods. For the healthy diet, these targets were derived from the serve sizes recommended in the New Zealand Eating and Activity Guidelines [29, 30] and the acceptable macronutrient distribution ranges, upper limit (sodium) and suggested dietary target (fibre) from the Nutrient Reference Values for Australia and New Zealand [31]. For the current diets, these targets were derived from average intakes reported in the nutrition surveys [19–22] and for sodium using a later survey which performed 24-h urine collection [32]. For current diets, about 30% of variation was allowed around the average population intakes for each of the targets, except for energy intake, where only 1.5% of variation was allowed.

- Healthy and current diet baskets

The common foods list was used to generate the list of foods in the current and healthy diet baskets. The current diet basket for the total population contained 100 foods (including takeaways, excluding alcohol), while the healthy diet basket contained 73 foods (excluding takeaways and alcohol). Takeaway items and discretionary foods were included in the standard current diets but not in the standard healthy diets. Alcohol was only included in the diets for specific scenarios.

Compared to the current diet, the healthy diet basket contained a higher variety of fruits and vegetables, healthier versions of common foods (e.g. canned tomatoes without added salt, some wholegrain or wholemeal products, low fat yoghurt) and a limit on the consumption of red meat (maximum 100 g per day). For each common food, minimum and maximum serve sizes were set based on nutrition survey data, to avoid unrealistic amounts of any one food in the generated meal plans.

#### Food composition data

Nutrient composition data and edible cooking factors for the common foods were used from the New Zealand Food Composition Database [33] and the New Zealand *Nutritrack* database of packaged food products [34].

#### Food prices data

Two sources of prices data were used:

- Prices were collected in spring (November 2016) in 12 Auckland supermarkets and their nearest fruit and vegetables store, in areas with different levels of deprivation. For each common food the cheapest price was collected, and the original price was also collected if the cheapest price was discounted. Prices of fruit and vegetables were collected in supermarkets as well as fruit and vegetable stores. In addition, for 6 out of 12 supermarkets, if the cheapest food was a generic item, the cheapest branded item was also collected. Based on consultations with Toi Tangata, a few common fruits and vegetables (e.g. feijoas, kamo kamo, puha, watercress) were included with zero cost for Māori, as these would always be gifted or gathered, not purchased. Based on advice from Pacific Heartbeat, for Pacific households prices were collected in 3 different additional supermarkets in South Auckland and fruit and vegetables were only priced from fresh produce markets.
- The Food Price Index (FPI) data for New Zealand [35] was used for the period 2007-2016 to examine trends in the cost of current New Zealand population diets over time. As some healthier options for certain food groups were not included in this dataset (e.g. low salt or low fat products, wholegrain foods, butter but no margarine), trends in cost of healthy diets were not assessed using the FPI. Items in the FPI are selected based on their expenditure in the Household Economic Survey. Prices are collected monthly from 56 supermarkets across 12 regional centres and from fresh fruit and vegetable stores, fish shops, butchers, convenience stores, restaurants and takeaway food outlets [35].

### Interface

The programme user interface [18] allows the user to specify the daily targets for the food groups and nutrients for all household members for current and healthy diets separately. In addition, the interface allows the user to specify whether or not to include takeaway meals, alcohol and discretionary foods as part of the diets.

The minimum serve size difference between any two generated individual meal plans was set at half a serve for any common food in this study.

**Fig. 1:**
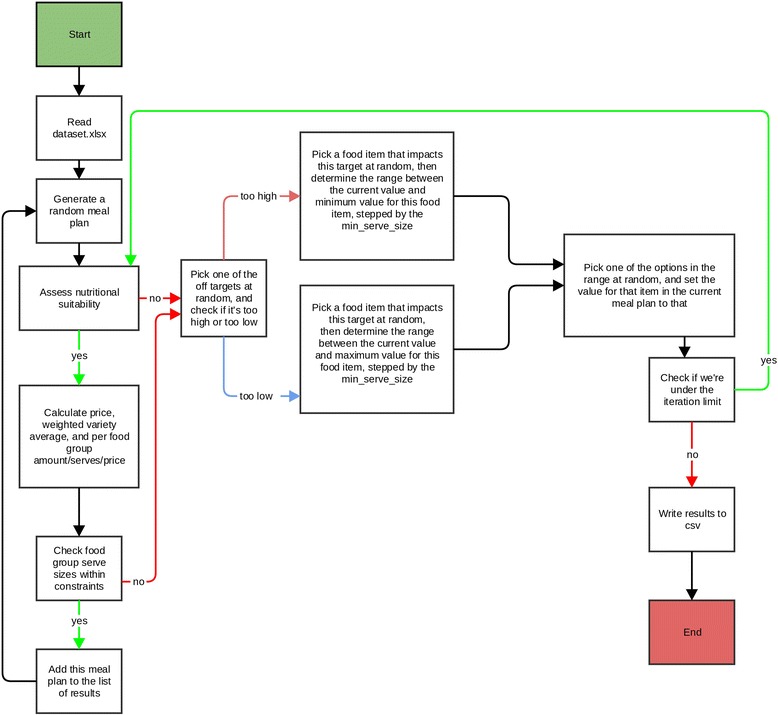
Algorithm of the DIETCOST program

The programme algorithm uses the Mersenne Twister as a random number generator to specify the starting meal plan and the starting value in grams for each of the common foods. If a meal plan meets all targets/constraints and is not already in the list of matching meal plans, it is added to the results. If it doesn’t (i.e. it fails some constraint), the algorithm will then try to fix that constraint (by raising/lowering the amount of some item that affects that constraint randomly between the min and max amount for that food item). If the modification results in a matching meal plan the meal plan is added to the results, and the same procedure starts again until the specified number of iterations has been run (Fig. 1). If the modification does not result in a matching meal plan, the algorithm will continue to try to resolve one of the failing constraints in a subsequent iteration. All success meal plans are independent from each other.

For each individual household member, the current and healthy diet scenarios were run multiple times with 1million, 2million and 20million iterations respectively to find the right number of iterations needed to accurately estimate the average cost of healthy and current household diets.

### Validation of menu plans

A random selection of meal plans (*N* = 8, 4 for the healthy and 4 for the current diets) for the different household members generated by the programme was validated by a research assistant who made fortnightly household meal plans with the same amount of common foods manually to make sure that the meal plans were acceptable. A few food items were linked within the programme to ensure the resulting meal plans are acceptable, e.g. the programme code specified that the total number of serves of milk needs to be higher than or equal to the total number of serves of breakfast cereals and the total number of serves of spreads equal or lower than the total number of serves of bread and crackers.

### Statistical analysis

All possible combinations of two-weekly meal plans for the four individual household members were assembled into two-weekly household diets for healthy and current diets separately. The range and distribution of the cost of the fortnightly household meal plans and the contributions of each food group and discretionary foods, alcohol and takeaways to the cost of the diets was calculated.

The impact of different prices, diets and policy scenarios on the cost differential between healthy and current household diets was also calculated.

## Results

About 1million iterations allowed an accurate estimation of the average cost of healthy household diets, while about 2million iterations were needed for an accurate estimation of the average cost of current household diets in New Zealand.

### Standard healthy and current diets for the total New Zealand population

The energy density of the average current household diet was about 50% higher than for the average healthy household diet. Current diets were all found less healthy than the healthy diets as, for example for the adult men, 0 meal plans met all healthy diet guidelines due to 0 meal plans meeting maximum sodium intake, minimum fibre intake and minimum number of serves of fruit (Additional file 1: Table S1).

**Fig. 2:**
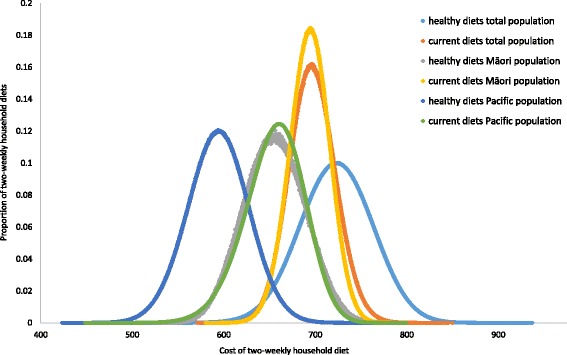
Distribution of the two-weekly cost of healthy (1,000,000 iterations) and current (2,000,000 iterations) household diets for the total New Zealand population and for Māori and Pacific population groups separately

The average fortnightly cost of healthy New Zealand household diets was about $27 more expensive than the average cost of current New Zealand household diets (Table 1). However, about 25.8% of healthy diets were cheaper than the cost of the average current household diets, and the cheapest healthy household diet was cheaper than the cheapest current diet (Fig. 2). The variation around the average cost was greater for healthy than for current diets and the range of costs of current diets fell within the range of costs of healthy diets (Table 1; Fig. 2).

**Table 1:** Average (SD) cost in New Zealand dollars and energy density of two-weekly standard household healthy and current, less healthy diets^a^ for the total New Zealand population and for Māori and Pacific population groups separately

	Total NZ population		Pacific population		Māori population	
	Healthy diets	Current diets	Healthy diets	Current diets	Healthy diets	Current diets
N iterations	1,000,000	2,000,000	1,000,000	2,000,000	1,000,000	2,000,000
N common foods included	73	100	71	105	75	109
N individual meal plans	365 + 347 + 319 + 137	360 + 185 + 96 + 153	120 + 226 + 299 + 281	252 + 452 + 330 + 329	93 + 137 + 17 + 44	332 + 127 + 12 + 367
N household meal plans	5,960,900,400	978,220,800	2,278,595,280	12,366,557,280	9,530,268	185,690,256
Average cost (SD)	$723.4 (75.7)	$696.3 (48.5)	$593.7 (65.0)	$655.1 (62.6)	$655.3 (65.0)	$693.6 (43.1)
Range of the cost	$502.0 - $937.1	$571.1 - $850.0	$423.4 - $778.3	$448.6 - $799.6	$522.0 - $790.7	$580.3 - $796.3
Energy (MJ)^b^	139.6 (137.6-141.7)	152.2 (150.3-154.8)	139.6 (137.6-141.7)	165.7 (163.8-168.7)	139.7 (137.6-141.4)	161.4 (159.4-164.1)
Energy density (kJ/g)^b^	1.02	1.49	1.03	1.56	0.94	1.65

*SD* standard deviation

^a^No alcohol, takeaways or discretionary foods included in the standard healthy diet; takeaways and discretionary foods included in the current diet

^b^Average (range)

**Fig. 3:**
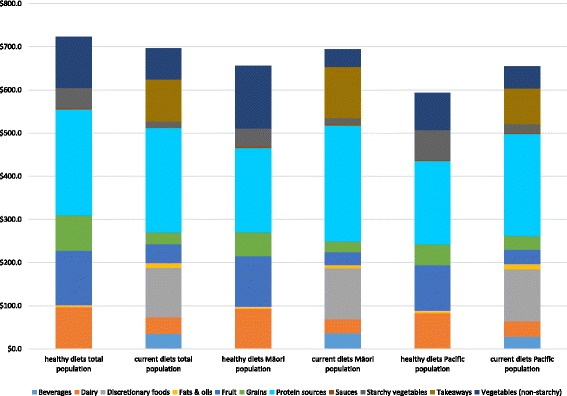
Contribution of food groups to the average cost of current and healthy diets for the total population and Māori and Pacific populations in New Zealand

Discretionary foods (including beverages) and takeaways contributed about 35.5% to the cost of the average current diet, while fruits and vegetables contributed about 19% to the average cost. Healthy diets did not contain takeaways and discretionary foods but fruits and vegetables contributed about 40% to the average cost of healthy diets. Protein sources were the major contributor to the cost of healthy and current diets and contributed on average more or less one third to the cost of both healthy and current diets, while dairy contributed about 5% to the cost of current versus 13% to the cost of healthy diets (Fig. 3).

### Diets for different ethnic population groups

When healthy and current household diets were modelled for specific ethnic New Zealand populations, fortnightly healthy household diets were less expensive on average than fortnightly current household diets; with an average differential of about $60 dollars for Pacific and $40 for Māori (Table 1). About 87.1% of healthy diets were cheaper than the average cost of current Māori diets, while 96.7% of healthy diets were cheaper than the average cost of current Pacific diets (Fig. 2). The current average energy intakes for both Pacific and Māori household members, based on the energy required to maintain the current BMI, were between 3 and 9% and 5-10% higher than for the total New Zealand population respectively (data not shown). The energy density of the current diets was highest on average for Māori households (Table 1).

Discretionary foods and takeaways contributed 35.5% and 39.3% to the average cost of Pacific and Māori current diets, while fruits and vegetables contributed 16% and 12% to the average cost respectively. Healthy diets did not contain takeaways and discretionary foods but fruits and vegetables contributed about 46% and 43% to the average cost of healthy diets for Māori and Pacific households respectively (Fig. 3).

### Diet, prices and policy scenarios

The average cost of the healthy diet was substantially more expensive for all population groups with inclusion of takeaways (healthier options such as sushi, sandwich) and substantially cheaper with an allowance for discretionary foods (Table 2). The latter was not the case for Māori populations since some fruit and vegetables are included in those diets for free (as Māori would never buy those foods). With an allowance for discretionary foods, the average cost of healthy diets was the same as the average cost of current diets for the total New Zealand population (Table 2). When allowing for the same energy intake in the healthy as in the current diet, the average cost of the healthy diet was $70 more expensive over a fortnight than the standard healthy diet and when allowing takeaways and alcohol at the same time, the average cost was $105 dollars more expensive over a fortnight (Table 2).

**Table 2:** Average (SD) cost in New Zealand dollars of two-weekly healthy and current, less healthy household diets^a^ for the total New Zealand population and for Māori and Pacific populations separately with and without allowance for takeaways, alcohol and discretionary foods

	Total NZ population		Pacific population		Māori population	
	Healthy diets	Current diets	Healthy diets	Current diets	Healthy diets	Current diets
Changes to the diets – scenarios						
Standard diets (as per Table 1)^a^	$723.4 (75.7)	$696.3 (48.5)	$593.7 (65.0)	$655.1 (62.6)	$655.3 (65.0)	$694.4 (49.1)
Including takeaways	$760.4 (81.7)	$696.3 (48.5)	$615.8 (74.2)	$655.1 (62.6)	$689.3 (70.7)	$694.4 (49.1)
Including alcohol and takeaways	$767.2 (85.6)	$720.5 (49.3)	$626.5 (75.6)	$677.2 (63.2)	$699.1 (78.4)	$712.4 (44.4)
Including discretionary foods	$697.8 (65.0)	$696.3 (48.5)	$576.7 (58.4)	$655.1 (62.6)	$659.7 (65.6)	$694.4 (49.1)
Including discretionary foods, alcohol and takeaways	$733.3 (70.3)	$720.5 (49.3)	$605.9 (59.1)	$677.2 (63.2)	$694.8 (83.7)	$712.4 (44.4)
Changes to the energy intake of the healthy diet - scenarios						
Same energy in current and healthy diet – without alcohol and takeaways	$793.9 (80.0)	$696.3 (47.9)	$707.3 (74.4)	$655.1 (62.6)	$777.8 (61.8)	$694.4 (49.1)
Same energy in current and healthy diet – with alcohol and takeaways	$828.7 (85.6)	$720.5 (49.3)	$758.4 (73.9)	$677.2 (63.2)	$798.7 (72.5)	$712.4 (44.4)

*SD* standard deviation

^a^No alcohol, takeaways or discretionary foods included in the healthy diet; takeaways and discretionary foods included in the current diet

When buying common foods at a discount whenever possible, the cost of the average healthy diet was about $14 per fortnight less expensive on average than when foods were not bought on discount. The same applies for buying generic products instead of brands, where the average healthy diet, when including generics, where available was $30 cheaper. Results for these scenarios were similar for current diets (Table 3).

**Table 3:** Comparison of the average (SD) cost in New Zealand dollars of two-weekly standard healthy and current, less healthy diets^a^ for the total New Zealand population for different pricing and policy scenarios

	Healthy diet	Current diet
Prices scenarios		
Non-discount prices only	$730.0 (75.8)	$700.2 (52.0)
Discount prices included	$716.4 (73.0)	$688.9 (48.4)
Prices for branded products only	$739.4 (73.6)	$712.4 (48.9)
Prices for generic products included	$706.0 (75.5)	$681.9 (48.9)
Fruit and vegetables from supermarkets	$738.5 (73.8)	$692.7 (48.9)
Fruit and vegetables from fresh produce stores	$706.3 (74.2)	$691.6 (47.7)
Policy scenarios		
Leaving GST off fresh fruit and vegetables^a^	$681.5 (72.2)	$671.0 (43.3)
Leaving GST off fresh fruit and vegetables and a 20% soda tax^a^	$681.5 (72.2)	$676.1 (43.5)

*SD* standard deviation, *GST* Goods and Services Tax

^a^No alcohol, takeaways or discretionary foods in the healthy diet; takeaways and discretionary foods included in the current diet

In the case where GST would be removed from fresh and frozen fruits and vegetables in New Zealand, such as in Australia, this would substantially reduce the cost differential between the healthy and current diets, to the extent that the average cost differential almost disappears. On the other hand, implementing a sugary drinks tax had only minimal impact on the cost differential between healthy and current diets in New Zealand, although it does reduce the average cost differential a bit further (Table 3).

### Trends in the cost of the New Zealand population diet over time

**Fig. 4:**
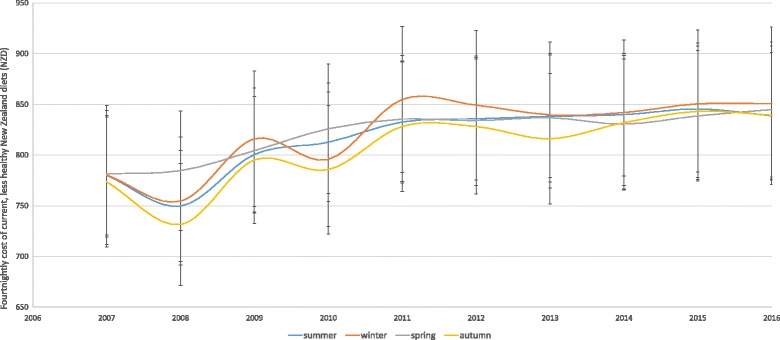
The average cost of current, less healthy diets over 10 years in New Zealand using the Food Price Index (FPI) prices in different seasons (summer, winter, spring and autumn)

Using the Food Price Index data over 10 years, the cost of current diets was a bit more expensive, since not all prices collected as part of the FPI are necessarily the cheapest prices. Based on this data, the cost of current household diets substantially increased over time. The contribution to the cost of different food groups was very similar across seasons though vegetables tend to contribute more to the cost in winter (Fig. 4, Additional file 1: Fig. S1).

## Discussion

This study developed a new tool and methodology, DIETCOST, to model the cost differential between healthy and current population diets, and applied it as a case study in New Zealand.

For the New Zealand population, healthy diets were on average $27 more expensive than current diets over a fortnight in New Zealand, but one-quarter of healthy diets were cheaper than the average current diet. For Māori and Pacific population groups, however, in view of their high current energy intakes, current household diets were on average $40-60 more expensive than healthy diets. Healthy diets for Māori and Pacific households are generally cheaper than for the total population because of several factors, e.g. prices in South Auckland supermarkets and fresh produce markets were cheaper (where Pacific populations live and shop), some fruits and vegetables, like feijoas, puha, kamo kamo, were included in the Māori diets for free since they would be gifted or gather these foods, and healthy Māori diets did not contain lower salt/fat/sugar versions of products.

Strengths of the study include the development and validation of a new programme, DIETCOST, to generate shopping lists for two-weekly meal plans that meet the targets and constraints for both the current and the healthy diets. In addition, common foods and nutrient and food group targets for different ethnic groups allowed conducting the modelling for specific ethnic populations. Unlike studies to date that have compared the cost of one healthy and one current diet, DIETCOST allows the cost of many fortnightly household diets to be generated enabling comparing the distribution of costs of current and healthy diets. DIETCOST provides a tool to calculate the cost of many meal plans for a range of scenarios of changing the diet contents, altering the type of price, location or reference household, without the need to do this manually. The programme can be readily used and applied with other population groups in other countries and contexts after adapting the input files. Limitations of the study include the fact that New Zealand lacks up-to-date nutrition survey data as the latest survey was conducted in 2008. A new nutrition survey needs to be conducted urgently and was a key recommendation in the latest Healthy Food Environment Policy Index report [36]. The Food Price Index is a great resource to look at trends over time, but includes some clear limitations, such as the fact that some key foods are missing and it is not always easy to divide the foods into healthy and less healthy due to insufficient details in the description.

This study adds to the literature through providing a common tool to assess the cost differential between current and healthy diets. Previous studies only priced one healthy and one current diet and used different approaches to identify the diet, define healthiness and calculate the cost [9].

In Australia, it was found that the current diet was more expensive than the healthy diet with more than half of the current household budget for food spent on energy-dense, nutrient-poor foods, which is more than in New Zealand [11]. It needs to be noted however that in Australia fruit and vegetables and basic healthy foods are exempt from GST. In addition, there was no adjustment for underreporting, while this study calculated energy intake based on the current weight of the population rather than using survey data. In Denmark, it was found that the healthy New Nordic diet was about 16% more expensive than the current diet with the largest relative difference for low-income households [37].

## Conclusions

The cost of food is an important determinant of food choices. This study developed a novel methodology to model the cost differential between healthy and current household diets. Healthy New Zealand diets are on average more expensive than current diets, but not for specific ethnic population groups.

Reducing taxes on fresh and frozen fruit and vegetables in New Zealand in conjunction with a sugary drinks tax could reduce the average cost of healthy diets towards the average of current diets and make it easier for people to consume healthy diets. The same approach as in this study can be applied in countries globally to benchmark the cost differential between current and healthy diets among countries internationally.

## Additional file

Additional file 1: Table S1. Proportion of current diets for adult males (*N* = 360) meeting the guidelines for a healthy diet. **Fig. S1.** Contributions of different food groups to the average cost of the current, less healthy diet in New Zealand by season (across 10 years). (DOCX 99 kb)

## Abbreviations

BMI: Body Mass Index
CDC: Centres for Disease Control
FPI: Food Price Index
GST: Goods and Services Tax
INFORMAS: International Network for Food and Obesity/NCDs Research, Monitoring and Action Support
NCDs: Non-communicable diseases
SSB: Sugar-sweetened beverages
