# Supplementary material for: Modelling the cost differential between healthy and current diets: the New Zealand case study
Source: Int J Behav Nutr Phys Act. 2018 Feb 9;15:16. doi: 10.1186/s12966-018-0648-6 (PMC5807767; doi:10.1186/s12966-018-0648-6)
Supplement: Supplementary file 1 — Table S1. Proportion of current diets for adult males (N = 360) meeting the guidelines for a healthy diet. Fig. S1. Contributions of different food groups to the average cost of the current, less healthy diet in New Zealand by season (across 10 years). (DOCX 99 kb) [file 12966_2018_648_MOESM1_ESM.docx]

**Additional file 1**

**Table S1** Proportion of current diets for adult males (N=360) meeting the guidelines for a healthy diet

| **Nutrient/Food Group** | **Guideline healthy diet per day** | **N (%)** |
| --- | --- | --- |
| Saturated fat (% energy) | 0-10 | 10 (2.8%) |
| Protein (% energy) | 15-25 | 352 (97.8%) |
| Discretionary foods (% energy) | 0% | 0 (0.0%) |
| Fibre (g) | 30 (minimum) | 0 (0.0%) |
| Sodium (mg) | 2300 (maximum) | 0 (0.0%) |
| Fat (% energy) | 20-35 | 16 (4.4%) |
| Carbohydrates (% energy) | 45-65 | 19 (5.3%) |
| Red meat (g) | 100 (maximum) | 282 (78.3%) |
| Dairy (serves) | 2 (minimum) | 0 (0.0%) |
| Vegetables (serves) | 2 (minimum) | 360 (100.0%) |
| Fruit (serves) | 2 (minimum) | 0 (0.0%) |
|  |  |  |
| All healthy diet guidelines |  | 0 (0.0%) |

**Figure S1** Contributions of different food groups to the average cost of the current, less healthy diet in New Zealand by season (across 10 years)
